# Cudratricusxanthone A Exhibits Antitumor Activities Against NSCLC Harboring EGFR L792H and G796R Triple Mutations via Regulating EGFR-ERK/AKT/STAT3 Signaling

**DOI:** 10.3390/molecules31091504

**Published:** 2026-04-30

**Authors:** Yinghao Wang, Jiamin Xian, Zhuoyi Wang, Jingmeng Wang, Ruohan Zhang, Jun Sheng, Jing Wang, Peiyuan Sun

**Affiliations:** 1 Key Laboratory of Development and Utilization of Food and Medicinal Resources, Ministry of Education, Yunnan Agricultural University, Kunming 650201, China; wyhh0518@163.com (Y.W.); xjm9798@163.com (J.X.); 18160229352@163.com (Z.W.); 15215003760@163.com (R.Z.); 2 Dabie Mountain Laboratory, College of Tea and Food Science, Xinyang Normal University, Xinyang 464000, China; 3 School of Medicine and Health, Guangdong Zhaoqing Aviation Vocational College, Zhaoqing 526107, China; 4 College of Science, Yunnan Agricultural University, Kunming 650201, China; 18087103497@163.com

**Keywords:** non-small cell lung cancer, EGFR L792H triple mutation, EGFR G796R triple mutation, cudratricusxanthone A, molecular mechanism

## Abstract

**Background:** Acquired resistance to the third-generation EGFR tyrosine kinase inhibitor osimertinib, often mediated by EGFR triple mutations, poses a major clinical challenge in non-small cell lung cancer (NSCLC) treatment. Among these, some rare mutations, such as L858R/T790M/L792H and L858R/T790M/G796R, create steric hindrance that directly interferes with osimertinib binding, yet effective targeted therapeutic strategies for these specific mutations remain lacking. Cudratricusxanthone A (CTXA), a natural xanthone derivative isolated from *Cudrania tricuspidata* Bur., has demonstrated various pharmacological activities, but its effects against EGFR triple-mutant NSCLC have not been systematically investigated. **Methods:** Stable Ba/F3 and NIH/3T3 cell lines expressing EGFR L858R/T790M/L792H or L858R/T790M/G796R triple mutations were generated via electroporation. The antiproliferative effects of CTXA were evaluated by MTT/MTS assays, colony formation, and wound healing assays. Cell cycle distribution and apoptosis were analyzed by flow cytometry. Protein expression of EGFR signaling pathway components (p-EGFR, p-ERK, p-AKT, p-STAT3) and cell cycle regulators (Cyclin D1, CDK4) were examined by Western blotting. Molecular docking and 200 ns molecular dynamics simulations were performed to investigate the stability and binding modes of CTXA to the mutant EGFR kinase domains. **Results:** The successfully established triple-mutant cell lines exhibited high EGFR expression, IL-3-independent growth, and significant resistance to osimertinib. CTXA inhibited the proliferation of all triple-mutant cell lines in a time- and concentration-dependent manner, with 48 h IC_50_ values ranging from 0.362 to 2.488 μM. Mechanistically, CTXA suppressed EGFR autophosphorylation and downregulated downstream p-ERK, p-AKT, and p-STAT3. CTXA induced G1 phase cell cycle arrest by downregulating Cyclin D1 and CDK4, significantly promoted apoptosis, and inhibited cell migration. Molecular docking revealed that while osimertinib binding was blocked by steric hindrance from His-792 or Arg-796, CTXA adapted to the mutated ATP-binding pockets through multiple hydrogen bonds and extensive hydrophobic interactions. Molecular dynamics simulations confirmed the stable binding of CTXA to both mutant EGFR proteins over the 200 ns simulations. **Conclusions:** This study demonstrates for the first time that the natural compound CTXA possesses antitumor efficacy against EGFR L858R/T790M/L792H and L858R/T790M/G796R mutants by regulating EGFR-ERK/AKT/STAT3 signaling. Our findings position CTXA as a promising lead compound for tackling this challenging form of acquired resistance and highlight the value of natural products in multi-target antitumor drug discovery.

## 1. Introduction

Lung cancer stands as the leading cause of cancer-related mortality worldwide, posing a major threat to human life expectancy. NSCLC constitutes approximately 85% of all lung cancer cases [1]. The pathogenesis and progression of NSCLC are closely linked to mutations in the epidermal growth factor receptor (EGFR) [2]. The advent of EGFR tyrosine kinase inhibitors (EGFR-TKIs) has significantly improved the prognosis for patients harboring sensitizing mutations, such as L858R and exon 19 deletions [3]. However, acquired resistance remains the primary challenge leading to treatment failure. Following the emergence of the secondary EGFR T790M mutation, which confers resistance to first- and second-generation TKIs, the introduction of the third-generation EGFR-TKI osimertinib has effectively overcome T790M-mediated resistance [4]. Nevertheless, patients often experience disease progression after approximately 10 months of treatment. A key mechanism underlying this progression is the development of triple mutations within the EGFR kinase domain, such as C797S, L792H, and G796R [5]. While significant research progress has been made regarding the C797S mutation [6], effective targeted therapeutic strategies are still lacking for the L792H and G796R mutations [7]. These mutations directly interfere with osimertinib binding through a steric hindrance effect, leading to treatment failure. To date, no commercial cell lines harboring these rare triple mutations are available, and no approved therapeutics exist for this patient subpopulation—representing a critical and unresolved clinical dilemma.

Natural products have consistently served as a vital source of lead compounds in antitumor drug discovery, owing to their chemical structural diversity, broad availability, and multi-target activities [8]. CTXA is a xanthone derivative isolated from the roots of the traditional medicinal plant *Cudrania tricuspidata* Bur [9]. Previous studies have reported that CTXA possesses various pharmacological activities, including anti-inflammatory, neuroprotective, and hepatoprotective effects, indicating its potential for multi-target modulation [10,11]. In our previous study, CTXA has been found to exert an inhibitory effect against NSCLC harboring wild-type EGFR by targeting EGFR [12]. However, whether CTXA exhibits activity against the EGFR L792H and G796R triple mutants remains unknown. Furthermore, the absence of available cell lines harboring these specific variants has hindered research into this area.

Recently, molecular docking and molecular dynamics simulations have gained prominence as the core methodologies in computer-aided drug design. They enable the prediction of binding modes, affinity, and interaction dynamics between a small molecule and a target protein at the atomic level, providing crucial theoretical insights for elucidating the mechanism of action of potential inhibitors [13]. In particular, molecular dynamics simulations serve to corroborate the outcomes of molecular docking simulations by providing critical dynamic characteristics of the target–ligand complex. This significantly enhances the accuracy of the computational models.

Therefore, in this study, Ba/F3 and NIH/3T3 cell lines stably expressing EGFR L858R/T790M/L792H and L858R/T790M/G796R mutations were generated and used to assess the activity of CTXA against these mutants. We systematically evaluated the effects of CTXA on the proliferation, migration, apoptosis, and cell cycle of these mutants by inhibiting the EGFR signaling pathway and its downstream cascades, including RAS/RAF/MEK/ERK, PI3K/AKT, and JAK/STAT3. Furthermore, we explored the interactions between CTXA and EGFR L792H and G796R mutants by molecular docking and molecular dynamics simulations. This study is expected to provide a novel candidate compound and mechanistic insights for overcoming osimertinib resistance.

## 2. Results

### 2.1. Establishment of EGFR L858R/T790M/L792H and L858R/T790M/G796R Mutant Cell Lines

**Figure 1:**
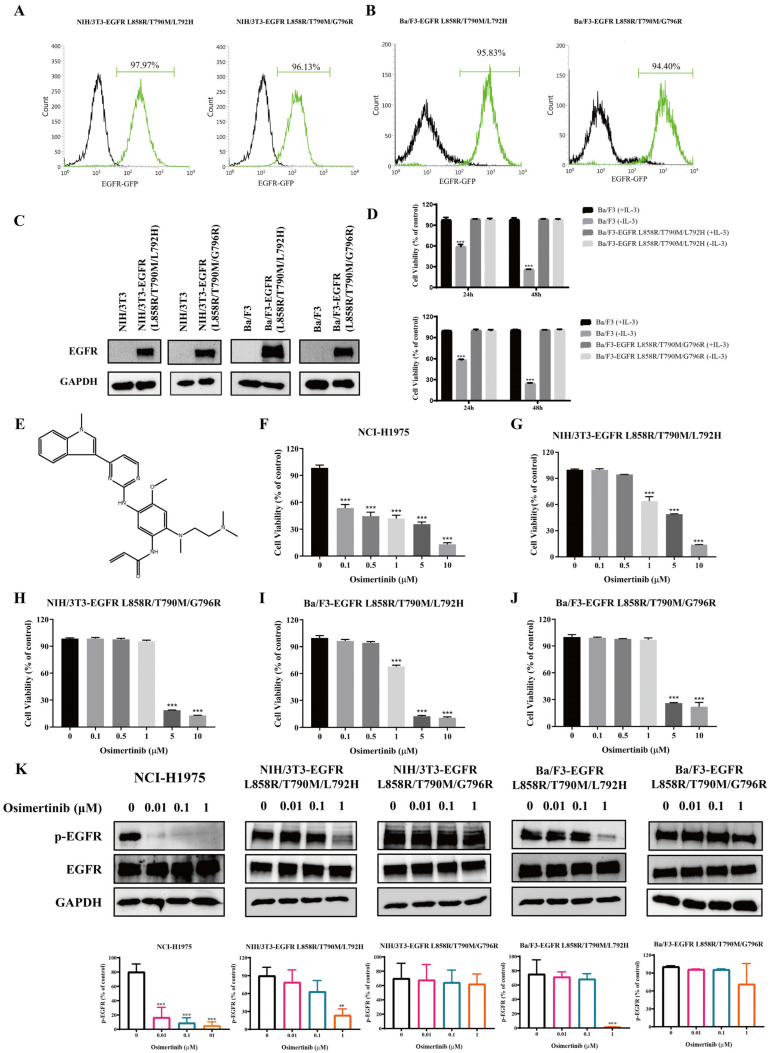
Establishment and characterization of EGFR L858R/T790M/L792H and L858R/T790M/G796R triple-mutant cell lines. (**A**,**B**) Flow cytometry analysis of EGFR expression in NIH/3T3 (**A**) and Ba/F3 (**B**) cells stably transfected with EGFR L858R/T790M/L792H or L858R/T790M/G796R mutations (Green). Untransfected parental cells served as negative controls (Black). (**C**) Western blot analysis confirming high expression of mutant EGFR proteins in the established triple-mutant cell lines. Parental NIH/3T3 and Ba/F3 cells showed no detectable EGFR expression. (**D**) IL-3 independence assay. Ba/F3 parental cells and Ba/F3-derived triple-mutant cells were cultured in the presence or absence of IL-3 for 24 and 48 h. Cell viability was measured by MTS assay. (**E**) The chemical structure of osimertinib. (**F**–**J**) Drug sensitivity assay. NCI-H1975 (**F**), NIH/3T3-EGFR L858R/T790M/L792H (**G**), NIH/3T3-EGFR L858R/T790M/G796R **(H**), Ba/F3-EGFR L858R/T790M/L792H (**I**), and Ba/F3-EGFR L858R/T790M/G796R (**J**) cells were treated with increasing concentrations of osimertinib (0–10 μM) for 24 h. Cell viability was determined by MTT (NIH/3T3) or MTS (Ba/F3) assays. NCI-H1975 cells (EGFR L858R/T790M) served as a positive control for osimertinib sensitivity. (**K**) Western blot analysis of p-EGFR (Tyr1068) expression in triple-mutant cells treated with osimertinib (0, 0.01, 0.1, and 1 μM) for 24 h. Total-EGFR and GAPDH were used as loading controls. Data are presented as the mean ± SD (*n* = 3). ** *p* < 0.01, *** *p* < 0.001 versus 0 group.

This study successfully established stable cell models expressing the EGFR L858R/T790M/L792H and L858R/T790M/G796R triple mutations. Four stable cell lines were constructed in both NIH/3T3 and Ba/F3 cells, respectively, through electroporation and monoclonal screening. Flow cytometry analysis revealed that the EGFR expression rate in each cell line exceeded 94% (Figure 1A,B). Western blotting further confirmed the high expression of mutant EGFR intracellularly (Figure 1C). Functional validation demonstrated that the Ba/F3-derived triple mutant cells were capable of normal proliferation in the absence of IL-3, indicating their growth factor-independent phenotype (Figure 1D). Drug sensitivity assays showed that all triple mutant cell lines exhibited significant resistance to the third-generation EGFR-TKI osimertinib: cell viability inhibition was less than 20% upon treatment with 0.1 μM osimertinib, and the inhibitory effect on p-EGFR in the G796R mutant remained negligible even at 1 μM concentration (Figure 1E–J). Western blot results further confirmed that low concentrations of osimertinib failed to effectively inhibit EGFR autophosphorylation in the triple-mutant cells (Figure 1K). These systematic validations indicate that the constructed cell models stably express EGFR triple mutations characterized by constitutive activation and osimertinib resistance, providing a reliable experimental platform for subsequent mechanistic studies and drug screening.

### 2.2. CTXA Inhibits the Proliferation and Viability of EGFR Triple-Mutant Cells

**Figure 2:**
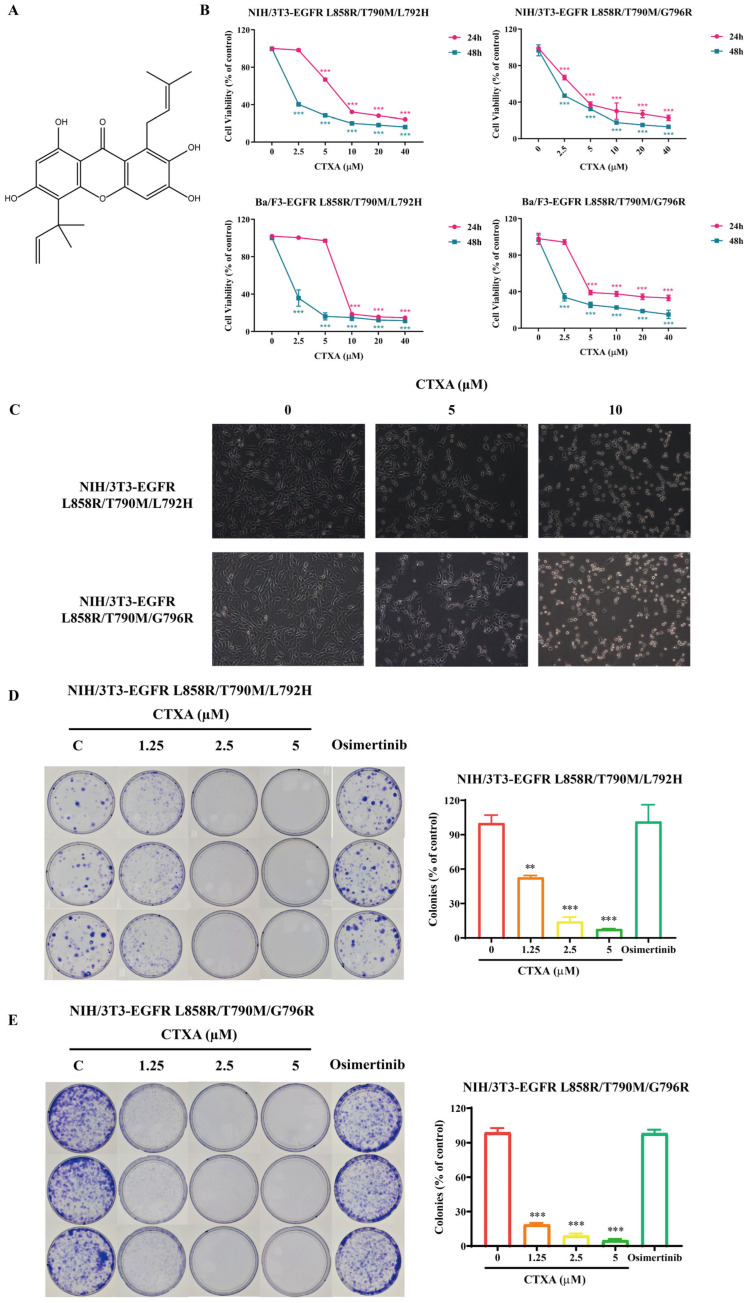
CTXA inhibits the proliferation and viability of EGFR triple-mutant cells. (**A**) The chemical structure of CTXA. (**B**) Cell viability assay. NIH/3T3 and Ba/F3 triple-mutant cells were treated with increasing concentrations of CTXA (0–40 μM) for 24 or 48 h. Cell viability was measured by MTT (NIH/3T3) or MTS (Ba/F3) assays. IC_50_ values at 48 h are presented in Table 1. (**C**) Morphological changes. NIH/3T3 triple-mutant cells were treated with CTXA (5 or 10 μM) for 6 h and observed under an inverted microscope. Scale bar = 200 μm. (**D**,**E**) Colony formation assay. NIH/3T3 triple-mutant cells were seeded at 1000 cells/dish and treated with CTXA (0, 1.25, 2.5, or 5 μM) or osimertinib (1 μM) for 10–14 days. Colonies were fixed, stained with crystal violet, and photographed. Data are presented as the mean ± SD (*n* = 3). ** *p* < 0.01, *** *p* < 0.001 versus control group.

To evaluate the inhibitory activity of CTXA against EGFR triple-mutant cells, its effects on the viability of different cell lines were first determined. MTT/MTS assays demonstrated that CTXA significantly inhibited the proliferation of all four triple-mutant cell lines in a time- and concentration-dependent manner (Figure 2A,B). The half-maximal inhibitory concentrations (IC_50_) at 48 h were 0.715 μM for Ba/F3-EGFR L858R/T790M/L792H cells, 0.362 μM for Ba/F3-EGFR(L858R/T790M/G796R) cells, 2.488 μM for NIH/3T3-EGFR L858R/T790M/L792H cells, and 1.973 μM for NIH/3T3-EGFR L858R/T790M/G796R cells (Table 1). At the morphological level (Figure 2C), microscopic observation after 6 h of CTXA treatment revealed that, starting from a concentration of 5 μM, cells exhibited typical death or apoptotic morphology, including significant shrinkage, rounding, and reduced optical transparency, in sharp contrast to the plump, well-adhered cells in the control group. Further colony formation assay results (Figure 2D,E) showed that CTXA markedly inhibited the long-term proliferative capacity of the triple-mutant cells. Even at lower concentrations (1.25 μM and 2.5 μM), the number and size of cell colonies in the CTXA-treated groups were significantly reduced compared to the control group. Treatment with 5 μM CTXA almost completely suppressed colony formation. Taken together, these results indicate that CTXA effectively inhibits the proliferation of cells harboring the EGFR L858R/T790M/L792H and L858R/T790M/G796R mutants.

### 2.3. CTXA Suppresses the EGFR Signaling Pathway in Triple-Mutant Cells

**Figure 3:**
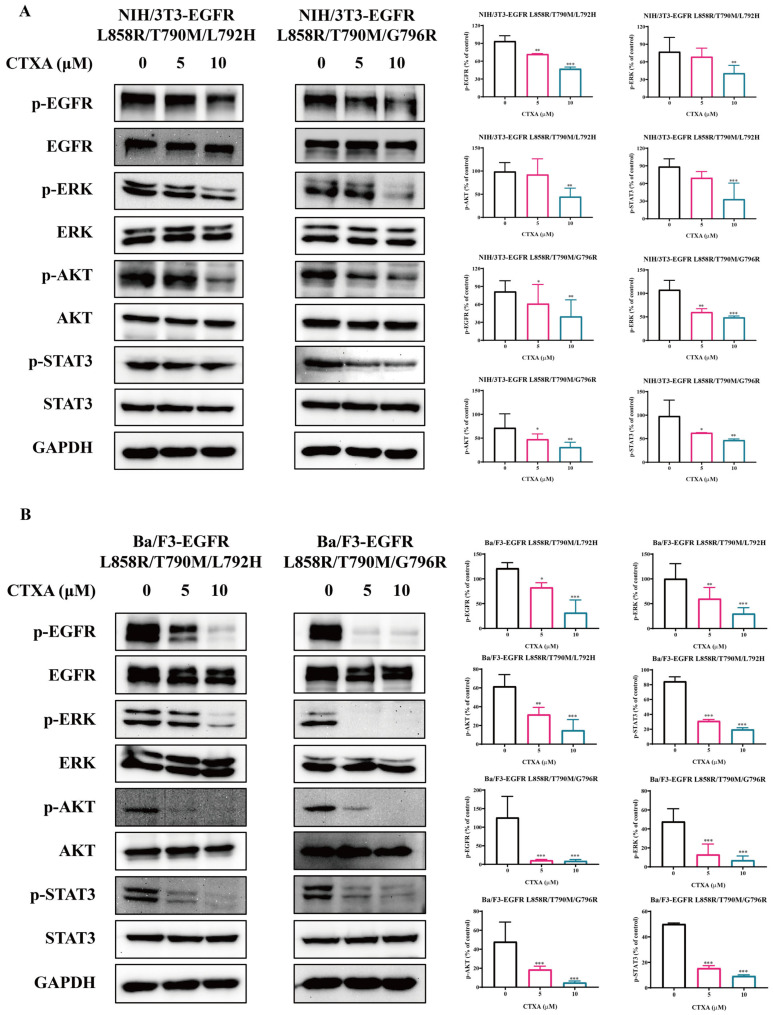
CTXA suppresses the EGFR signaling pathway in triple-mutant cells. (**A**,**B**) Western blot analysis of EGFR signaling pathway proteins. NIH/3T3 (**A**) and Ba/F3 (**B**) triple-mutant cells were treated with CTXA (0, 5, or 10 μM) for 1 h. Total cell lysates were immunoblotted with antibodies against p-EGFR, total EGFR, p-ERK, total ERK, p-AKT, total AKT, p-STAT3, total STAT3, and GAPDH. Quantitative analysis of protein expression levels. The relative phosphorylation levels of EGFR, ERK, AKT, and STAT3 were quantified by densitometry and normalized to their respective total protein levels. Data are presented as the mean ± SD (*n* = 3). * *p* < 0.05, ** *p* < 0.01, *** *p* < 0.001 versus 0 group.

To investigate the molecular mechanism by which CTXA inhibits the proliferation of triple-mutant cells, we examined its effects on the phosphorylation levels of EGFR and its key downstream signaling proteins via Western blotting. As shown in Figure 3A (NIH/3T3) and Figure 3B (Ba/F3), CTXA treatment significantly downregulated the level of EGFR autophosphorylation (p-EGFR) in both cell lines. Concurrently, the phosphorylation levels of pivotal downstream nodes including extracellular signal-regulated kinase (p-ERK), protein kinase B (p-AKT), and signal transducer and activator of transcription 3 (p-STAT3) also exhibited a concentration-dependent decrease. Quantitative analysis indicated that treatment with 10 μM CTXA produced the most pronounced inhibitory effect on all the aforementioned phosphorylated proteins, while 5 μM CTXA was sufficient to cause a marked downregulation of most proteins. These results suggest that CTXA can effectively block survival and proliferation signaling in triple-mutant cells by inhibiting the activation of EGFR and its downstream ERK, AKT, and STAT3 pathways.

### 2.4. CTXA Induces G1 Phase Cell Cycle Arrest in Triple-Mutant Cells

**Figure 4:**
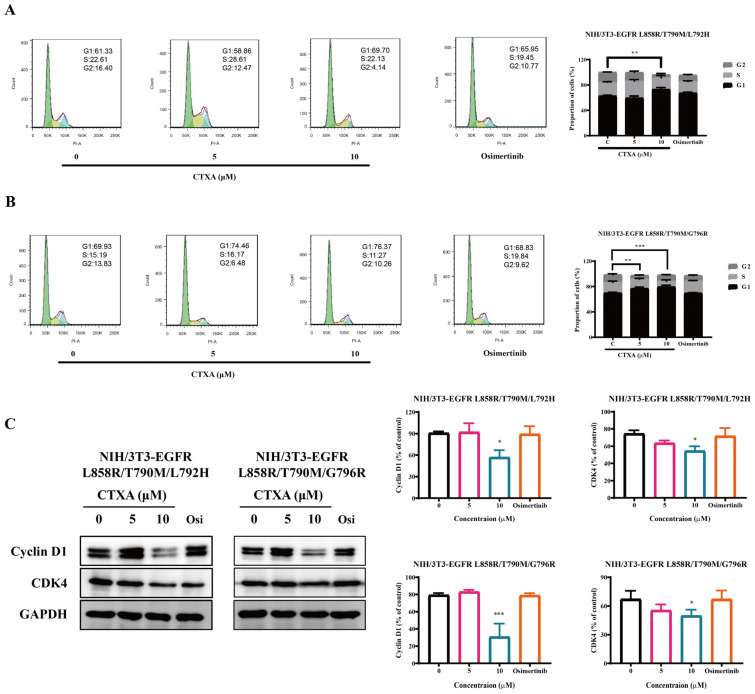
CTXA induces G1 phase cell cycle arrest in EGFR triple-mutant cells. (**A**,**B**) Cell cycle distribution analysis. NIH/3T3 triple-mutant cells harboring L858R/T790M/L792H (**A**) or L858R/T790M/G796R (**B**) mutations were treated with CTXA (0, 5, or 10 μM) or osimertinib for 24 h. Cell cycle distribution was analyzed by flow cytometry using propidium iodide (PI) staining. In the representative histograms (left panels), G1, S, and G2/M phases are shown in green, yellow, and blue, respectively. Quantitative analysis of the percentage of cells in G1, S, and G2/M phases (right panels) are shown. Data are presented as the mean ± SD (*n* = 3). ** *p* < 0.01, *** *p* < 0.001 versus control group. (**C**) Western blot analysis of cell cycle-related proteins. NIH/3T3 triple-mutant cells were treated with CTXA (0, 5, or 10 μM) or osimertinib for 24 h. Total cell lysates were subjected to immunoblotting with antibodies against Cyclin D1, CDK4, and GAPDH. Data are presented as the mean ± SD (*n* = 3). * *p* < 0.05, *** *p* < 0.001 versus control group.

To elucidate the potential mechanism underlying the inhibition of cell proliferation by CTXA, we further investigated its impact on cell cycle progression. Flow cytometry analysis revealed that CTXA treatment significantly altered the cell cycle distribution of NIH/3T3 triple-mutant cells, as evidenced by a concentration-dependent increase in the proportion of cells in the G1 phase and a corresponding decrease in cells in the S and G2/M phases (Figure 4A,B). This effect was not observed in the osimertinib-treated group. Consistent with this phenotype, Western blotting results showed that CTXA downregulated the expression levels of Cyclin D1, a key regulator of the G1/S transition, and its dependent kinase CDK4 in a concentration-dependent manner (Figure 4C). These results demonstrate that CTXA induces G1 phase arrest in EGFR triple-mutant cells by inhibiting the Cyclin D1/CDK4 pathway, thereby suppressing cell cycle progression and proliferation.

### 2.5. CTXA Induces Apoptosis and Inhibits Migration in Triple-Mutant Cells

**Figure 5:**
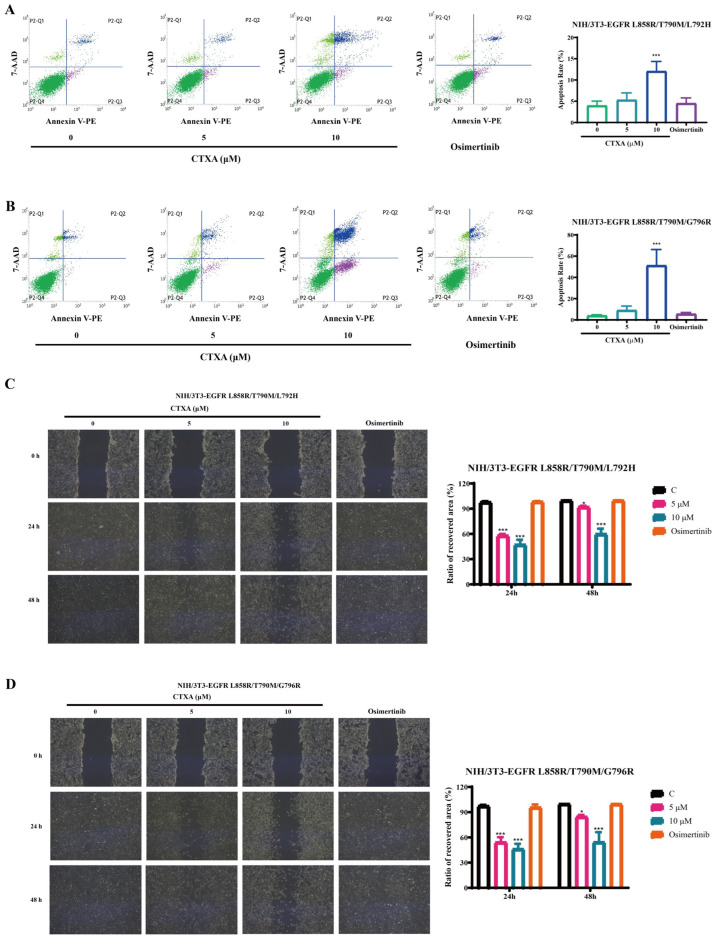
CTXA induces apoptosis and inhibits migration in EGFR triple-mutant cells. (**A**,**B**) Apoptosis analysis. NIH/3T3 triple-mutant cells harboring L858R/T790M/L792H (**A**) or L858R/T790M/G796R (**B**) mutations were treated with CTXA (0, 5, or 10 μM) or osimertinib for 24 h. The colors indicate the following: green, normal cells; purple, early apoptotic cells; blue, late apoptotic cells; and light green, dead cells. Apoptosis was detected by flow cytometry using Annexin V-PE/7-AAD double staining. Data are presented as the mean ± SD (*n* = 3). *** *p* < 0.001 versus 0 group. (**C**,**D**) Wound healing migration assay. NIH/3T3 triple-mutant cells were grown to confluence, scratched with a sterile pipette tip, and treated with CTXA (0, 5, or 10 μM) or osimertinib (1 μM) for 24 and 48 h. (**C**) Representative images of wound closure at 0, 24, and 48 h are shown. (**D**) The wound closure rate was quantified using ImageJ 1.52 software. Data are presented as the mean ± SD (*n* = 3). * *p* < 0.05, *** *p* < 0.001 versus control group.

To further evaluate the antitumor activity of CTXA, we investigated its effects on two critical malignant phenotypes: apoptosis and migration. Flow cytometry analysis with Annexin V-PE/7-AAD double staining revealed that CTXA significantly induced apoptosis in a concentration-dependent manner. Treatment with 10 μM CTXA increased the apoptotic ratio to approximately 15% in L858R/T790M/L792H mutant cells and to as high as approximately 50% in L858R/T790M/G796R mutant cells (Figure 5A,B), suggesting that the G796R mutant cells are more sensitive to the pro-apoptotic effect of CTXA. Meanwhile, wound healing assay results demonstrated that CTXA effectively inhibited the migration ability of the triple-mutant cells. At both 24 and 48 h, the wound closure rates in CTXA-treated groups were significantly lower than those in the control group, and the inhibitory effect exhibited both concentration and time dependence (Figure 5C,D). In contrast, osimertinib treatment showed no significant impact on apoptosis or migration. These results collectively indicate that CTXA exerts its antitumor effects at multiple levels, not only by directly killing cells through the induction of apoptosis but also by inhibiting their migratory capacity.

### 2.6. Molecular Docking Reveals the Structural Basis for CTXA Overcoming Osimertinib Resistance

**Figure 6:**
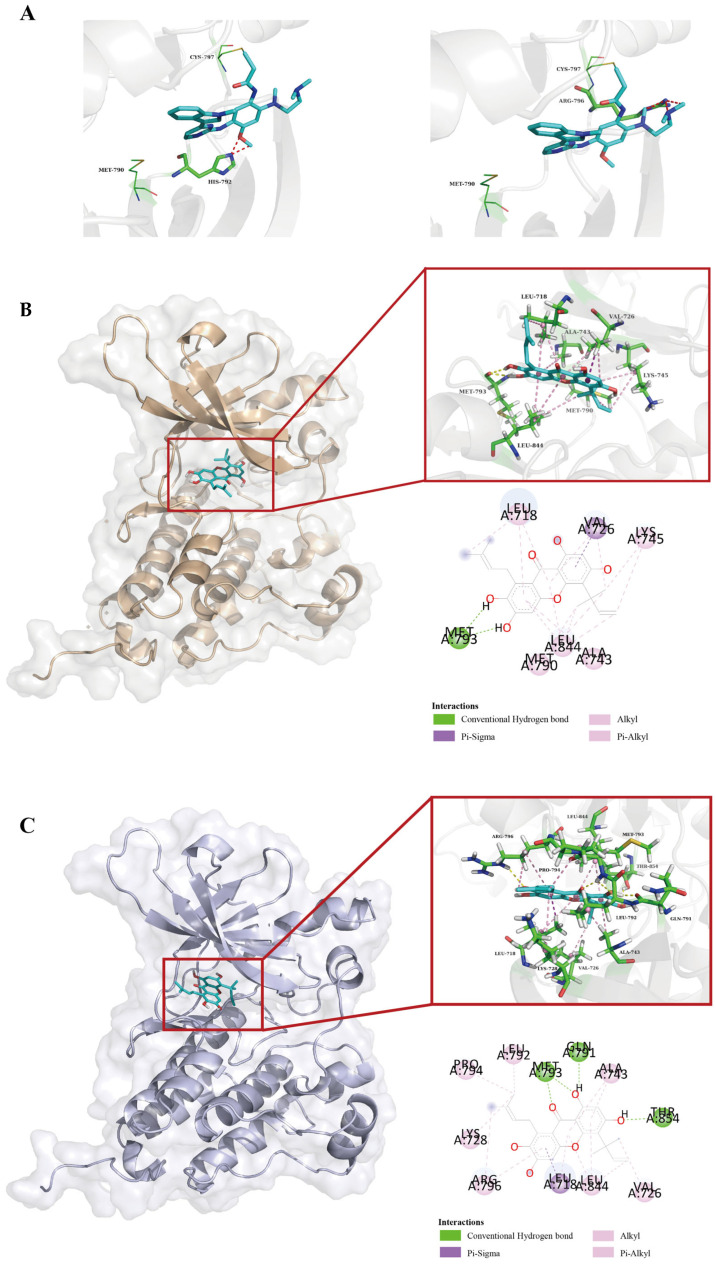
Molecular docking reveals the structural basis for CTXA overcoming osimertinib resistance. (**A**) Molecular docking analysis of osimertinib binding to EGFR T790M/L792H (left) and T790M/G796R (right) mutant kinase domains. The imidazole ring side chain of His-792 (L792H) and the long guanidinium group side chain of Arg-796 (G796R) create steric hindrance (red dashed lines) that blocks osimertinib binding. (**B**) Molecular docking analysis of CTXA binding to EGFR T790M/G796R mutant kinase domain. CTXA forms hydrophobic interactions (Alkyl/Pi-Alkyl) with Leu844, Lys745, Leu718, Ala743, Met790, and Val726; a conventional hydrogen bond with Met793; and a Pi-sigma interaction with Val726. (**C**) Molecular docking analysis of CTXA binding to the EGFR T790M/L792H mutant kinase domain. CTXA forms hydrophobic interactions (Alkyl/Pi-Alkyl) with Pro794, Leu844, Leu718, Lys728, Val726, Ala743, and Met793; a Pi-sigma interaction with Leu718; and conventional hydrogen bonds with Arg796, Leu792, and Gln791.

To explore the potential mechanism by which CTXA overcomes osimertinib resistance at the structural level, we performed molecular docking simulation studies. Absent the crystal structures of the EGFR L792H and G796R mutants, the EGFR kinase domain containing the T790M mutation was used as the template for mutating Leu792 to His or Gly796 to Arg. As shown in Figure 6A, upon the occurrence of the L792H and G796R mutations, the imidazole ring side chain of histidine (His792) in the L792H mutation and the large side chain along with the hydrophilic group of arginine (Arg796) in the G796R mutation both created significant steric hindrance against the aniline-pyrimidine core structure of osimertinib and its acrylamide group. This provides a structural explanation for the failure of osimertinib in such triple-mutant cells.

In contrast, CTXA was able to effectively bind to the ATP-binding pocket of both mutant proteins in a distinct manner (Figure 6B,C). The binding energy of CTXA to the L792H and G796R mutant proteins was −5.61 and −7.16 kcal/mol, respectively. For the G796R mutant, CTXA primarily engaged via hydrophobic interactions (Alkyl/Pi-Alkyl) with multiple residues including Leu844, Lys745, Leu718, Ala743, Met790, and Val726. It also formed a conventional hydrogen bond with Met-793 and exhibited a Pi-sigma interaction with Val726. For the L858R/T790M/L792H mutant protein, CTXA similarly bound through extensive hydrophobic interactions (Alkyl/Pi-Alkyl) with residues such as Pro794, Leu844, Leu718, Lys728, Val726, Ala743, and Met793, and established a Pi-sigma interaction with Leu718. Crucially, CTXA was able to form multiple conventional hydrogen bonds with Arg796, Leu792, and Gln791.

In summary, the molecular docking simulations indicate that osimertinib resistance is directly related to the steric hindrance induced by the triple mutations. By forming multiple hydrogen bonds and extensive hydrophobic interactions, the xanthone scaffold of CTXA can effectively adapt to the active pockets of both triple-mutant EGFR kinase domains. This provides a rational structural basis for its ability to overcome osimertinib resistance at the cellular level.

### 2.7. Molecular Dynamics Simulations Validate the Stable Binding of CTXA to EGFR L792H and G796R Mutants

**Figure 7:**
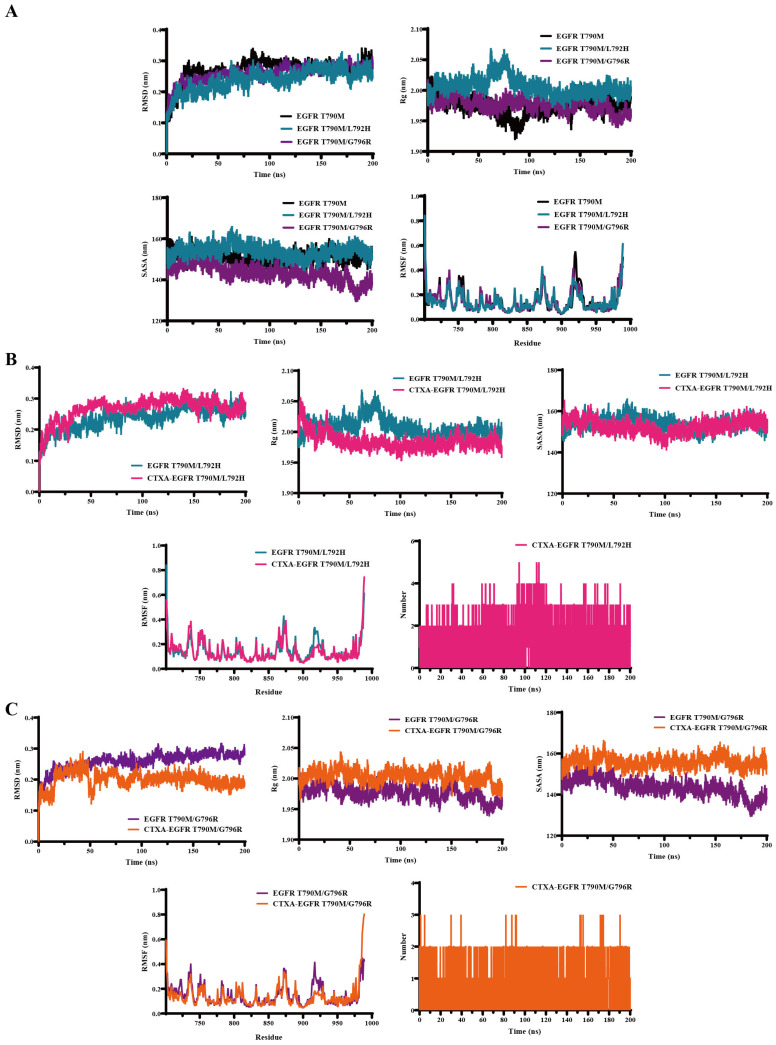
Molecular dynamics simulations validate the stable binding of CTXA to EGFR proteins with L792H or G796R mutations. (**A**) Dynamics characteristics of EGFR T790M kinase domain upon occurring the L792H or G796R mutations. (**B**,**C**) Molecular dynamics simulation analysis of CTXA binding to EGFR T790M/L792H (**B**) and T790M/G796R (**C**) mutant kinase domains over 200 ns simulations. Key stability parameters are shown: RMSD and Rg of free proteins and CTXA-EGFR complexes, RMSF of amino acid residues, number of hydrogen bonds formed between CTXA and the proteins, and SASA of the binding pocket. The simulations demonstrate rapid convergence and stable maintenance of protein–ligand complexes throughout the simulation period.

To thoroughly evaluate the binding stability of CTXA with the two EGFR mutant proteins (L792H and G796R) at a dynamic level, molecular dynamics (MD) simulations lasting 200 ns were conducted. Key parameters, including root mean square deviation (RMSD), radius of gyration (Rg), root mean square fluctuation (RMSF), number of hydrogen bonds, and solvent accessible surface area (SASA), were systematically analyzed. As shown in Figure 7A, the simulation system reached equilibrium after 100 ns. After the occurrence of the L792H or G796R mutation, the RMSD values of the two mutant proteins was comparable to those of the EGFR T790M mutant, suggesting that L792H and G796R mutations did not markedly compromise the overall structural stability. However, the G796R mutation obviously rendered the protein structure more compact, which may further explain why the G796R triple mutant is more resistant to osimertinib than the L792H triple mutant.

Throughout the simulation period, for both complex systems formed by CTXA and the mutant proteins, the RMSD values (Figure 7B,C) rapidly reached equilibrium within a low range and remained stable thereafter. This result clearly indicates that CTXA forms stable complexes within the ATP-binding pockets of both mutants, with no significant fluctuations in the overall conformation of the complexes, robustly confirming their stability in a dynamic environment.

RMSF analysis revealed that upon binding of CTXA to the two EGFR triple-mutant proteins, the mobility of amino acid residues in key active loop regions of the kinase domain was effectively reduced. This phenomenon suggests that CTXA binding positively contributes to maintaining the conformational stability of this functional region. From a structural dynamics perspective, this hints at a potential sustained inhibitory effect on kinase activity, providing important clues for investigating the mechanism by which CTXA affects kinase function. Monitoring of the number of hydrogen bonds showed that stable hydrogen bond interactions between CTXA and both proteins were maintained throughout the 200 ns simulation. This not only strongly validates the reliability of the docking predictions but also indicates that hydrogen bonding is a key molecular foundation for the stable binding of CTXA to the proteins, explaining the origin of complex stability from the perspective of chemical forces. Interestingly, CTXA induced a more relaxed (less compact) conformation of the G796R mutant, as reflected by increased Rg and SASA values. Consistent with our cellular data, this may account for the higher antitumor activity of CTXA in the G796R triple mutant compared to the L792H triple mutant.

In summary, systematic analysis of multiple key parameters fully confirms that CTXA can maintain a stable complex structure at a dynamic level when binding to both EGFR triple-mutant proteins, L858R/T790M/L792H and L858R/T790M/G796R. This provides a solid structural dynamics foundation for in-depth research into the antitumor mechanism of CTXA based on targeting EGFR.

## 3. Discussion

**Figure 8:**
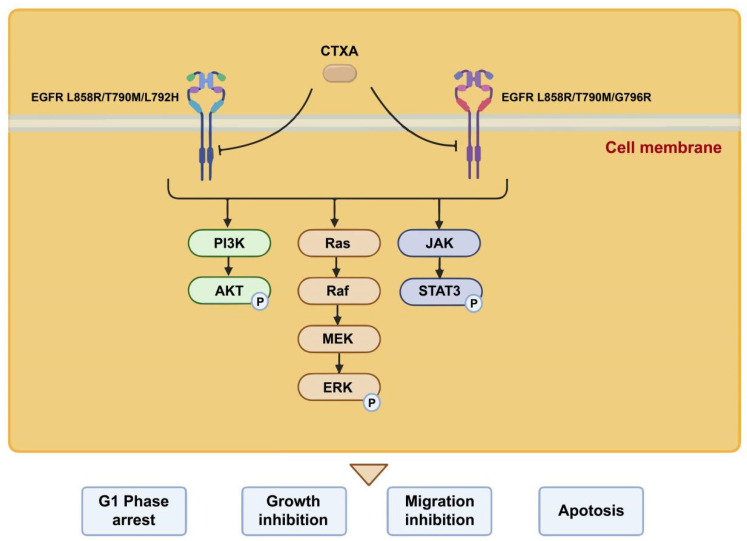
The anticancer mechanisms of CTXA against EGFR L858R/T790M/L792H and L858R/T790M/G796R mutants.

The emergence of acquired resistance mediated by tertiary EGFR mutations represents a pivotal clinical challenge in the targeted therapy of non-small cell lung cancer [14]. While third-generation EGFR-TKIs like osimertinib effectively overcome resistance conferred by the secondary T790M mutation, the subsequent development of triple mutations, such as C797S, L792H, and G796R, leads to treatment failure [4,15,16,17]. Notably, rare mutations like L792H and G796R introduce bulky side chains that create direct steric hindrance within the ATP-binding pocket of the kinase domain, physically blocking the covalent binding of osimertinib, which constitutes a well-defined mechanism of primary resistance [7,18]. While significant efforts are being made to develop strategies against steric hindrance-based resistance (e.g., to C797S) [19], effective and approved therapeutic options specifically for mutations like L792H and G796R remain lacking, representing a persistent gap in precision oncology. Therefore, the discovery of novel compounds capable of circumventing this steric hindrance to effectively inhibit such mutant kinases retains urgent clinical significance [20]. Here, we address this unmet need by identifying and systematically characterizing the natural xanthone derivative CTXA, demonstrate its potent inhibitory activity against NSCLC harboring EGFR L858R/T790M/L792H and L858R/T790M/G796R triple mutations in vitro and elucidate its multi-layered mechanisms of action and potential binding mode, thereby providing a valuable lead compound and a theoretical foundation for developing new strategies to overcome this form of resistance. The molecular mechanisms are summarized in Figure 8.

To establish a relevant disease model, we first engineered stable cell lines expressing these two EGFR triple mutations. This model successfully recapitulated the overexpression of mutant EGFR and the consequent growth-factor-independent proliferation. Crucially, it fully replicated the primary resistance phenotype to osimertinib observed clinically, which aligns perfectly with the hypothesized steric hindrance mechanism caused by L792H or G796R mutation [7,18]. This highly relevant model provided a solid platform for subsequent compound screening and mechanistic validation under stringent conditions. Our activity screening revealed that CTXA effectively inhibited the proliferation of both triple-mutant cell lines in a clear concentration- and time-dependent manner, with 48 h half-maximal inhibitory concentration values in the low micromolar range (0.362–2.488 μM). The significant inhibitory effect of CTXA, in stark contrast to the loss of activity of osimertinib in these cells, suggests that its mechanism of action is distinct from the traditional binding mode susceptible to steric hindrance, indicating unique potential to overcome this specific resistance.

At the molecular level, CTXA exhibits a hallmark multi-target, multi-pathway intervention profile, which may partly explain its efficacy against complex resistance mechanisms. Upstream in receptor signaling, CTXA treatment significantly suppressed the autophosphorylation of EGFR at key tyrosine residues (e.g., Tyr1068) and subsequently downregulated the activation of pivotal downstream nodes, including p-ERK, p-AKT, and p-STAT3. Sustained STAT3 activation has been identified as a key common mediator of acquired resistance to EGFR-TKIs, often arising from bypass signaling or feedback loops [21]. The effective suppression of p-STAT3 by CTXA implies its potential to disrupt this critical pro-survival axis, thereby overcoming or delaying the survival advantage of resistant cells. The precise regulation of the cell cycle is a fundamental target for anticancer therapeutics, with its dysregulation being a hallmark of cancer. Key proteins governing the G1/S phase transition, particularly the Cyclin D1-CDK4/6 complex, serve as critical checkpoints whose inhibition can halt uncontrolled proliferation. Targeting this axis has emerged as a validated strategy in cancer therapy, as evidenced by the clinical application of CDK4/6 inhibitors [22,23,24]. Regarding cell fate determination, CTXA induced G1 phase cell cycle arrest by downregulating the expression of Cyclin D1 and its dependent kinase CDK4, core positive regulators of the G1/S checkpoint, thereby curbing aberrant proliferation at its root. Furthermore, CTXA demonstrated a potent direct pro-apoptotic capability, significantly increasing the proportion of both early and late apoptotic cells. Concomitantly, it markedly inhibited cell migration and invasion—critical parameters in evaluating anticancer efficacy, as suppressing metastatic potential is essential for controlling disease progression [25]. These three facets of activity—inhibiting growth signals, arresting the cell cycle, and inducing programmed cell death while limiting metastatic potential—are not isolated effects but constitute a coordinated, multi-pronged antitumor strategy. This highlights the distinctive advantage of natural products in multi-target modulation, a recognized feature that may help to mitigate the bypass resistance frequently associated with single-target inhibition [26].

To elucidate the drug–target interaction underlying CTXA’s efficacy, we employed well-established computational approaches. Molecular docking and molecular dynamics simulations are powerful tools for predicting binding modes and assessing interaction stability at atomic resolution, providing critical insights into mechanisms of drug action and resistance [27,28]. Applying these methods, our molecular docking results visually elucidated the structural basis of osimertinib’s failure against the triple mutants: the imidazole ring side chain of His792 (L792H) or the long guanidinium group of Arg796 (G796R) directly invades and occupies the binding space of osimertinib, preventing it from adopting its active conformation. In stark contrast, these simulations revealed that CTXA achieves stable binding within the altered ATP-pocket, forming a complementary network of interactions that bypasses the steric clash. This predicted binding mode directly correlates with and provides a mechanistic explanation for the potent inhibitory effects observed in our cellular assays, effectively linking the compound’s atomistic behavior to its phenotypic outcome. In sharp contrast, the rigid planar xanthone scaffold of CTXA, coupled with its flexible substituents, can effectively adapt to the topologically altered ATP-binding pocket following mutation. Analysis indicated that CTXA is primarily anchored within the pocket through extensive van der Waals and alkyl-π interactions with hydrophobic residues such as Leu718, Val726, Ala743, Met790, and Leu844—a pattern of hydrophobic stabilization crucial for high-affinity binding in drug–target complexes [29]. Simultaneously, hydroxyl and carbonyl groups on the CTXA molecule form a directed hydrogen-bond network with key polar residues, including Gln791, Met793, and Arg796. This binding mode, characterized by hydrophobic anchoring supplemented by specific hydrogen bonds, appears to achieve a complementary fit with the spatial alterations induced by the mutations. Subsequent molecular dynamics simulations further corroborated that the CTXA-mutant EGFR complexes exhibited rapid convergence and maintained stability in key metrics like protein–ligand RMSD and critical interactions throughout the 200-nanosecond simulations, a robust approach for validating docking predictions and assessing binding stability [30]. This integrated computational analysis dynamically supports the rationality of the predicted binding mode and provides a solid structural biology basis for explaining its biological activity at the cellular level.

In conclusion, this study is the first to reveal that the natural xanthone compound CTXA potently inhibits the growth of NSCLC cells driven by EGFR L858R/T790M/L792H and L858R/T790M/G796R triple mutations. Its antitumor activity is achieved through a combination of synergistic mechanisms: effectively inhibiting EGFR autophosphorylation and its downstream ERK/AKT/STAT3 signaling axis, inducing G1 phase cell cycle arrest, and activating apoptosis while suppressing cell migration. Integrated with molecular docking and dynamics simulations, we provide, for the first time, structural-level insight demonstrating that CTXA can effectively adapt to the spatial conformation of the mutant kinase domain by forming multiple hydrogen bonds and extensive hydrophobic interactions, thereby circumventing the steric hindrance effect caused by L792H/G796R mutations. This offers direct molecular evidence for its ability to overcome osimertinib resistance. Based on the results of this present study, further experiments are warranted in the next step, including evaluation of in vivo efficacy, pharmacokinetics, toxicity evaluation, and ultimately clinical validation. Taken together, these findings may provide a novel therapeutic option for NSCLC harboring the L858R/T790M/L792H or L858R/T790M/G796R triple mutation.

## 4. Materials and Methods

### 4.1. Reagents and Compounds

Cudratricusxanthone A (>96.5% purity) was obtained from Yunnan Xili Biotechnology Co., Ltd. (Kunming, China). Osimertinib (AZD9291, >99.8% purity) was purchased from TargetMol (Boston, MA, USA). Dimethyl sulfoxide (DMSO), purchased from VWR Life Science (Atlanta, GA, USA), was used to dissolve CTXA and osimertinib for stock solutions stored at −20 °C. Epidermal Growth Factor (EGF) was purchased from Sino Biological (Beijing, China). Puromycin and hygromycin were obtained from Solarbio (Beijing, China). 3-(4,5-dimethylthiazol-2-yl)-2,5-diphenyltetrazolium bromide (MTT) and MTS reagent were purchased from Solarbio (Beijing, China) and Promega (Madison, WI, USA), respectively. All primary antibodies (EGFR, p-EGFR Tyr1068, ERK, p-ERK, AKT, p-AKT Ser473, STAT3, p-STAT3 Tyr705, Cyclin D1, CDK4, GAPDH) were purchased from Cell Signaling Technology (Danvers, MA, USA) or ABclonal Technology (Wuhan, China). All secondary antibodies were purchased from Thermo Fisher Scientific (Waltham, MA, USA).

### 4.2. Cell Lines and Culture

Mouse embryonic fibroblast NIH/3T3 cells and mouse pro-B Ba/F3 cells were used in this study. NIH/3T3 cells were cultured in Dulbecco’s Modified Eagle Medium (DMEM, MeilunBio, Dalian, China). Ba/F3 cells were maintained in Roswell Park Memorial Institute (RPMI) 1640 medium (MeilunBio, Dalian, China). All media were supplemented with 10% fetal bovine serum (FBS, Gibco, Waltham, MA, USA) and 1% penicillin/streptomycin (MeilunBio, Dalian, China). Parental Ba/F3 cells were cultured with interleukin-3 (IL-3) supplied from WEHI-3 cell supernatant. All cells were maintained in a humidified incubator with 5% CO_2_ at 37 °C.

### 4.3. Establishment of Stable EGFR Triple-Mutant Cell Lines

Plasmids carrying the EGFR L858R/T790M/L792H or L858R/T790M/G796R mutations, along with puromycin (for Ba/F3) or hygromycin (for NIH/3T3) resistance genes, were transfected into respective parental cells via electroporation using an Invitrogen Neon system. Transfected cells were selected and maintained in medium containing 2 μg/mL puromycin or 200 μg/mL hygromycin, supplemented with 10 ng/mL EGF. Stable monoclonal cell lines were isolated by limiting dilution in 96-well plates. EGFR expression was confirmed by flow cytometry (BD FACSVerse, BD Biosciences, San Jose, CA, USA) using an EGFR-FITC antibody (Santa Cruz Biotechnology, Dallas, TX, USA) and by Western blotting.

### 4.4. Cell Viability Assay

Cell viability was assessed using MTT (for NIH/3T3) or MTS (for Ba/F3) assays [31]. Cells were seeded in 96-well plates. After 24 h, cells were treated with serial dilutions of CTXA or osimertinib for 24 or 48 h. MTT or MTS reagent was then added according to the manufacturer’s protocol. The absorbance was measured at 492 nm (MTT) or 490 nm (MTS) using a microplate reader (BioTek, Agilent Technologies, Santa Clara, CA, USA). The half-maximal inhibitory concentration (IC_50_) was calculated using GraphPad Prism 7.0 software.

### 4.5. Colony Formation Assay

NIH/3T3 triple-mutant cells were seeded into 60 mm culture dishes at a density of 1000 cells per dish. After 24 h, cells were treated with different concentrations of CTXA or DMSO (control). The medium containing the compound was refreshed every 72 h. After 10–14 days, colonies were fixed with methanol, stained with 0.01% crystal violet, and photographed. The dye was dissolved in 10% acetic acid, and the absorbance at 560 nm was measured to quantify colony formation [32].

### 4.6. Western Blotting

Cells were lysed using RIPA buffer (Solarbio, Beijing, China) containing PMSF. Protein concentration was determined by a BCA assay. Equal amounts of protein were separated by SDS-PAGE and transferred onto PVDF membranes (Millipore, Burlington, MA, USA). After blocking, membranes were incubated with primary antibodies overnight at 4 °C, followed by incubation with HRP-conjugated secondary antibodies. Protein bands were visualized using a FluorChem E detection system (ProteinSimple, Santa Clara, CA, USA). Quantification was performed using ImageJ 1.52 software.

### 4.7. Cell Cycle Analysis

NIH/3T3 triple-mutant cells were treated with compounds for 24 h, harvested, and fixed in 70% cold ethanol overnight at 4 °C. Cells were then stained with a propidium iodide (PI) solution containing RNase A (4A Biotech, Beijing, China). Cell cycle distribution was analyzed using a flow cytometer [33] (BD FACSVerse, Franklin Lakes, NJ, USA), and data were processed with FlowJo V10 software.

### 4.8. Cell Apoptosis Assay

Apoptosis was detected using an Annexin V-PE/7-AAD apoptosis detection kit [34] (MeilunBio, Dalian, China). NIH/3T3 triple-mutant cells were treated with compounds for 24 h, collected, and stained according to the manufacturer’s instructions. The percentages of apoptotic cells were quantified by flow cytometry (BD FACSVerse, Franklin Lakes, NJ, USA).

### 4.9. Wound Healing Assay

A wound healing assay was used to analyze cell migration affected by CTXA [35]. NIH/3T3 triple-mutant cells were seeded in 6-well plates and grown to confluence. A straight scratch was created using a sterile 200 μL pipette tip. After washing, cells were incubated with serum-free medium containing compounds. Images of the wound area were captured at 0, 24, and 48 h under an inverted microscope. The wound closure rate was analyzed using ImageJ 1.52 software.

### 4.10. Computational Molecular Docking and Dynamics Simulations

The three-dimensional structure of CTXA was downloaded from PubChem (https://pubchem.ncbi.nlm.nih.gov). Due to the lack of the crystal structures for the EGFR L792H and G796R mutants, the crystal structure of the EGFR kinase domain harboring the T790M mutation (PDB: 6JX0) was obtained from the Protein Data Bank (https://www.rcsb.org) and used as the template. Subsequently, DeepView 4.1.0 was used to introduce the mutations (L792H and G796R) and minimize energy. Molecular docking was performed with AutoDock 4.2.6 to predict binding poses, and then the docking poses with the lowest binding energy were analyzed and visualized by Pymol 1.3 and Discovery Studio 2024 [36].

Subsequently, molecular dynamics simulations were conducted for the complexes using the GROMACS 2020.6 package [37]. The force field of AMBER99SB and the water model of SPC were set to assign all proteins. Following 200 ns of molecular dynamics simulations, key stability parameters, including root mean square deviation (RMSD), radius of gyration (Rg), solvent-accessible surface area (SASA), hydrogen bond numbers, and root mean square fluctuation (RMSF), were analyzed.

### 4.11. Statistical Analysis

All experiments were performed independently at least three times. Data are presented as the mean ± standard deviation (SD). Statistical significance between groups was determined using *t*-test or one-way analysis of variance (ANOVA) with GraphPad Prism 8.0. Differences were considered statistically significant at * *p* < 0.05, ** *p* < 0.01, and *** *p* < 0.001.

## 5. Conclusions

In summary, our study demonstrates that CTXA exerts antitumor activity against EGFR L858R/T790M/L792H and L858R/T790M/G796R mutants by inhibiting cell proliferation and migration, inducing G1 phase arrest, and promoting cell apoptosis. These effects are mediated through the direct interactions of CTXA with the EGFR L792H and G796R mutants, which leads to the suppression of EGFR activation and its downstream signaling molecules including ERK, AKT, and STAT3. These findings will provide a prospective candidate for treating NSCLC harboring EGFR L792H and G796R triple mutations.

## Figures and Tables

**Table 1:** The IC_50_ values of CTXA on various cells.

Cell Line	IC_50_ of 24 h (µM)	IC_50_ of 48 h (µM)
Ba/F3-EGFR L858R/T790M/L792H	8.063	0.715
Ba/F3-EGFR L858R/T790M/G796R	8.015	0.362
NIH/3T3-EGFR L858R/T790M/L792H	8.836	2.488
NIH/3T3-EGFR L858R/T790M/G796R	4.047	1.973

## Data Availability

Data will be available from the corresponding author on reasonable request.

## Abbreviations

The following abbreviations are used in this manuscript:

NSCLC	Non-small cell lung cancer
EGFR	Epidermal growth factor receptor
AKT	Protein Kinase B
CDK4	Cyclin-Dependent Kinase 4
ERK	Extracellular Signal-Regulated Kinase
GAPDH	Glyceraldehyde-3-Phosphate Dehydrogenase
IL-3	Interleukin-3
MD	Molecular dynamics
MTT	3-(4,5-Dimethylthiazol-2-yl)-2,5-Diphenyltetrazolium Bromide
MTS	3-(4,5-Dimethylthiazol-2-yl)-5-(3-Carboxymethoxyphenyl)-2-(4-Sulfophenyl)-2H-Tetrazolium
RMSD	Root mean square deviation
RMSF	Root mean square fluctuation
SASA	Solvent accessible surface area
Rg	Radius of gyration
